# Salivary Gland Dysfunction in Stroke Patients Is Associated with Increased Protein Glycoxidation and Nitrosative Stress

**DOI:** 10.1155/2020/6619439

**Published:** 2020-12-10

**Authors:** Mateusz Maciejczyk, Piotr Gerreth, Anna Zalewska, Katarzyna Hojan, Karolina Gerreth

**Affiliations:** ^1^Department of Hygiene, Epidemiology and Ergonomics, Medical University of Bialystok, 2C Adama Mickiewicza Street, 15-022 Bialystok, Poland; ^2^Private Dental Practice, 57 Kasztelanska Street, 60-316 Poznan, Poland; ^3^Experimental Dentistry Laboratory, Medical University of Bialystok, 24A Marii Sklodowskiej-Curie Street, 15-276 Bialystok, Poland; ^4^Department of Rehabilitation, Greater Poland Cancer Centre, 15 Garbary Street, 61-866 Poznan, Poland; ^5^Department of Occupational Therapy, Poznan University of Medical Sciences, Swiecickiego Street 6, 60-781 Poznan, Poland; ^6^Department of Risk Group Dentistry, Chair of Pediatric Dentistry, Poznan University of Medical Sciences, 70 Bukowska Street, 60-812 Poznan, Poland

## Abstract

Stroke is one of the leading causes of disability and death worldwide. Despite intensive medical care, many of the complaints directly threatening the patient's life marginalize their dental needs after the stroke. Recent studies indicate reduced saliva secretion in stroke patients in addition to the increased incidence of caries and periodontal disease. Since oxidative stress plays a vital role in the pathogenesis of salivary gland hypofunction and neurodegenerative disorders (including stroke), this is the first to evaluate the relationship between salivary gland activity and protein glycoxidation and nitrosative damage. The content of glycation and protein oxidation products and nitrosative stress was assessed in nonstimulated (NWS) and stimulated (SWS) whole saliva of stroke patients with normal salivary secretion and hyposalivation (reduced saliva production). The study included 30 patients in the stroke's subacute phase and 30 healthy controls matched by age and sex. We have shown that stroke patients with hyposalivation show increased contents of protein glycation (↑Amadori products and ↑advanced glycation end products), glycoxidation (↑dityrosine), and nitration (↑nitrotyrosine) products compared to stroke cases with normal salivary secretion and control group. Interestingly, higher oxidative/nitrosative stress was found in NWS, which strongly correlates with salivary flow rate, total protein content, and salivary amylase activity. Such relationships were not observed in the control group. Summarizing, oxidative and nitrosative stress may be one of the mechanisms responsible for the impairment of saliva secretion in stroke patients. However, extraglandular sources of salivary oxidative stress in stroke patients cannot be excluded. Further studies to assess salivary gland hypofunction in stroke cases are necessary.

## 1. Introduction

Stroke is a severe health problem in the modern world. It is defined as sudden, focal, vascular damage to the central nervous system confirmed by the presence of a stroke focus in neuroimaging studies or persistence of focal symptoms for more than 24 hours [1]. The most important risk factors for the disease are hypertension, heart disease, diabetes, dyslipidemia, and clotting disorders, which occur in most stroke patients [1]. Cerebrovascular accident is the third leading cause of severe long-term disability, the second dominant reason for death, and a fundamental cause of depression and dementia [2]. Although stroke has mainly vascular pathology, the disease affects both the brain vessels and the entire body. Indeed, stroke-related disorders include limb paresis, headaches, epileptic seizures, deep vein thrombosis, or urinary tract infections. The incidence of stroke complications is high and affects 40-96% of hospitalized or rehabilitated patients [3].

A key role in stroke pathogenesis is attributed to oxidative stress (OS) [3, 4]. OS is defined as an oxidative-reductive imbalance (in favor of oxidation reactions) depending on lipid raft turnover, mitochondrial function, and cellular cross-talk in the neurovascular coupling [5, 6]. Excessive formation of reactive oxygen species (ROS) generates oxidative damage to various macromolecules (i.e., proteins, lipids, and DNA), changing several signaling pathways and promote cellular injury and death [5, 7]. In ischemic stroke, excess calcium, sodium, and ADP levels are responsible for the overproduction of mitochondrial free radicals. In contrast, in hemorrhagic stroke, ROS sources are mainly the conversion of arachidonic acid to prostanoids and degradation of hypoxanthine [8, 9]. Cerebral oxidative stress also exaggerates the inflammatory response (by induction of the NfkB signaling pathway), leading to enhanced expression of cytokines, chemokines, and growth factors [10]. However, nitric oxide (NO) also plays a vital role in stroke complications. It cytotoxically damages the DNA, blocks the mitochondrial function, and intensifies free radical damage by peroxynitrite formation [11].

In our previous study, we showed that in stroke patients, enhanced OS occurs not only in the blood but also in saliva [12]. Disturbances of enzymatic and nonenzymatic antioxidant barrier and increased oxidative damage to salivary lipids were noted in both nonstimulated (NWS) and stimulated whole saliva (SWS) of stroke cases. In these patients, oral hygiene and periodontal conditions are also markedly worse than age- and gender-matched healthy control [13]. This condition predisposes to other oral diseases such as burning mouth syndrome, xerostomia (subjective dry mouth), or fungal infections [14]. Nevertheless, it is still unknown whether stroke patients suffer from OS-mediated hyposalivation. This hypothesis is likely since oxidative stress is the primary factor damaging the salivary glands in both oral and systemic diseases [15, 16]. Indeed, protein glycoxidation products can aggregate and accumulate in the salivary glands leading to morphological changes and qualitative and quantitative disorders of saliva secretion [17–19]. However, of all cellular biomolecules, proteins are believed to be the primary target of ROS attack [20]. Oxidizing agents are responsible for the oxidation of single amino acid residues or entire polypeptide chains and the formation of cross-linking and tearing of the protein chain. Interestingly, oxidatively modified proteins reduce or lose their biological activity and inhibit the mechanisms responsible for their degradation [21]. Nevertheless, reduced NO bioavailability (in the secretory cells of the salivary glands) may also result in abnormalities in the protein secretion into saliva [22]. OS-mediated salivary hypofunction has been demonstrated in patients with obesity [23, 24], hypertension [22], chronic kidney disease [16], chronic heart failure [25], psoriasis [26], but also neurodegenerative diseases such as dementia and Alzheimer's disease [27, 28]. Thus, OS could be a common denominator for central (brain) and local (salivary glands) complications in stroke patients.

Our study is aimed at assessing the relationship between salivary gland function/protein secretion into saliva and protein glycoxidation and nitrosative damage. The content of glycation and protein oxidation products and nitrosative stress was evaluated in NWS and SWS of stroke patients with normal salivary secretion and hyposalivation.

## 2. Material and Methods

### 2.1. Ethical Issues and Patient's Consent

Before the study, it was approved by the Bioethics Committee of the Poznan University of Medical Sciences (resolutions 59/19 and 890/19).

All participants, i.e., stroke patients and healthy controls, were provided with information concerning the research's purpose, procedures, benefits, and risks. Full written consent was obtained from all patients in accordance with the Declaration of Helsinki for dental examination and sampling of saliva.

### 2.2. Study Subjects

The study was performed between June and September 2019 in a health center (Bonifraterskie Centrum Zdrowia) in Piaski–Marysin (Piaski, Poland). The health center hospitalizes patients with various disorders, including those after cerebral stroke, from different country provinces.

The participation of each individual in the study was voluntary. One experienced neurorehabilitation specialist qualified all the individuals for the study according to its criteria.

The study group consisted of stroke patients in the subacute phase who were emerged out of 385 individuals that were subjects in the neurorehabilitation ward following different incidents, such as spinal cord injury, brain injury, vascular brain damage, surgically treated patients with a brain tumor, myelopathy, polyneuropathy, and sclerosis multiplex. It was established that 253 (65.71%) patients were stroke survivors. They were admitted to the neurorehabilitation unit directly from the hospital, in a subacute stroke phase, immediately after the acute phase cessation. Most individuals were able to communicate, cooperate, and understand instructions. A medical doctor assessed each patient, and subsequently, he/she was subjected to comprehensive individual and similar rehabilitation. Moreover, most individuals followed the same diet divided into a baseline diet for most people or a diet for diabetes mellitus patients. All the meals were distributed to the patients at the same time daily and were prepared in this hospital.

Data concerning the condition and general health status of individuals were obtained from patients' files and contained: age, gender, time since diagnosis of cerebral stroke, medical history, and medication used.

In addition, for measurement of the functional status of the patient, the following scales were utilized:

1. Addenbrooke's Cognitive Examination III (ACE III)—to differentiate individuals without and with cognitive impairment [29]
2. The Berg Balance Scale (BBS) for determining an individual's inability or ability to safely balance during a series of predetermined tasks [30]
3. The Barthel Index (BI) for measurement of performance in activities of daily living (ADL) [31]
4. The functional independence measure (FIM) explores the patient's physical, psychological, and social functioning [32]

Finally, 30 (11.86% of stroke survivors; 7.79% of all rehabilitated patients at the health center) fully completed the examination and were considered in the analysis (Table 1). The other individuals were excluded since, as many as 48 (12.47%) people were uncooperative because they could not communicate and give conscious written informed consent for participation in the research. Additionally, 117 (30.39%) individuals declined to participate in the study, and 34 (8.83%) patients did not come for examination and saliva sampling, even though they gave informed consent and were reminded 3–4 times. Additionally, 14 (3.64%) subjects resigned from the research after sampling of nonstimulated saliva due to physiological or psychological tiredness, 7 (1.82%) people were unable to sample the saliva because of general difficulties in understanding the procedure due to cognitive and language deficits, and 3 (0.78%) patients were taken to the other hospital because of deterioration of general health.

Patients from the study group (*n* = 30) were divided into two subgroups considering the rate of salivary secretion: NS—normal salivary secretion (*n* = 16), HS—reduced salivary secretion (hyposalivation; *n* = 14). NWS flow below 0.2 mL/min was considered as hyposalivation [16, 22, 33]. All participants from the control group (*n* = 30) had NWS flow > 0.2 mL/min.

### 2.3. Study Criteria

The inclusion criteria of stroke patients to the study were as follows: confirmed cerebral infarction or cerebral hemorrhage based on CT and magnetic resonance imaging (MRI); recovery from the acute phase of ischemic or hemorrhagic stroke in all brain areas; good general condition; consciousness and giving of written and informed consent for saliva sampling and oral examination; the age of consent (>18 years); first admission to cure stroke unit was more than 5–6 (to 10) hours from the onset of the early neurological symptoms; adequate capacity to follow instructions, i.e., being able to answer questions during the examination, understanding how to perform the procedures and ability to collect a saliva sample.

The exclusion criteria of subjects from the research were as follows: unconfirmed cerebral infarction or cerebral hemorrhage with CT and magnetic resonance imaging (MRI); stroke recurrence during subacute phase; poor general condition; unconsciousness and inability to give informed consent for saliva sampling and oral check-up; insufficient cooperation due to cognitive/language deficits; incapability to collect saliva sample; patients under the age of 18; legal guardianship; ischemic stroke treated with thrombectomy or thrombolysis; patients with psychiatric or cognitive disorders; lung disease (chronic obstructive pulmonary disease) or cardiovascular disease (angina or uncontrolled hypertension); autoimmune disease (rheumatoid arthritis, systemic lupus erythematosus); heart failure resting oxygen saturation (SaO_2_) ≤ 92%; women suffering from malnutrition, i.e., having body mass index lower than 18 kg/m^2^ or with weight loss over 10% during the previous three months. In addition, individuals taking dietary supplements and vitamins for the last three months and smokers were excluded from the research.

### 2.4. Control Group

The control group contained 30 individuals similar to the study group regarding gender and age, comorbidities, dentition and periodontium status, and oral hygiene (Table 1). Controls included patients reporting for dental check-ups to the Department of Restorative Dentistry of the Medical University of Bialystok (Bialystok, Poland) between March and September 2017. All individuals were provided with medical clearance by clinicians before involvement in the study. Subjects followed a regular, balanced diet (not restricted), and they were given standard physical activity recommendations.

### 2.5. Saliva Sampling

The sampling of saliva was carried out in the health center during summer time, i.e., between June and September, to keep similar weather conditions outside.

The research material was total mixed nonstimulated saliva (NWS) and stimulated saliva (SWS), and both types of these oral bioliquid samples were collected via spitting. The saliva was collected between 7 : 30 a.m. and 9 : 00 a.m. from patients who had restrained from intensive physical activity for the preceding twelve hours. Subjects were instructed not to intake any liquid and/or solid food other than clean water at least two hours before sampling of saliva. The individuals were also indicated not to perform any oral hygiene practices (i.e., teeth brushing, gum chewing, and mouth rinsing). Because all patients were in the subacute phase of stroke, they had to take medicines approximately eight hours before saliva sampling, but the time from the last dose of any medication was minimally two hours. The controls had not taken any medication eight hours before saliva sampling [27]. The saliva was gathered in a separate, private room after a five-minute adaptation to the environment. Before this oral bioliquid was collected, the oral cavity was rinsed two times with distilled water at room temperature to avoid possible contamination from other sources. During the study, the individuals tried to limit their face and lips' movements, and they were seated in an adjustable chair, individually adapted to the height of each patient, with the head slightly bent downwards and resting in a convenient position. The saliva samples which were gathered during the first minute were ejected. Saliva was collected into a sterile Falcon tube situated in a container with ice (temperature of approximately 0°C). The nonstimulated saliva was gathered for 10 minutes to avoid the patients' physiological and/or psychological tiredness, whereas SWS was collected in the same manner for 5 minutes. The saliva secretion was stimulated by the use of 10 *μ*L of 2% citric acid on the central part of the tongue every 30 seconds [12, 33–36]. Subsequently, after saliva sampling, this oral bioliquid volume was measured using a calibrated pipette with an accuracy of 0.1 mL [34]. The minute flow of nonstimulated and stimulated saliva was evaluated by dividing the bioliquid volume by the time necessary for its secretion and expressed in mL/min. Instantly, after sampling of saliva, it was centrifuged (+4°C, 20 min, 3000 × g; MPW 351, MPW Med. Instruments, Warsaw, Poland). Butylated hydroxytoluene (BHT, Sigma-Aldrich, Saint Louis, MO, USA) was added to the acquired supernatants, in the amount of 10 *μ*L 0.5 M BHT in acetonitrile (ACN)/1 mL of saliva, to protect the samples from oxidation processes [37]. The saliva samples were frozen at −80°C and stored for no more than three months for subsequent analysis.

### 2.6. Oral Examination

The dental examination was carried out in a separate room, shortly after saliva sampling. According to the World Health Organization criteria, the dentition was evaluated in artificial lighting, using a plane mouth mirror and a dental probe [38]. The patient's check-up was performed while seated in a chair with his/her head resting against the wall and with the dentist positioned in front of the chair. Each tooth was evaluated and scored as healthy, decayed (DT), extracted because of the carious process (MT), or filled due to caries (FT). The data gathered were utilized to calculate the DMFT index, which is the sum of DT, MT, and FT, and it expresses dental caries experience. The prevalence of dental caries was calculated as a percentage of patients with DMFT > 0. Plaque index (PlI) and Gingival index (GI) were also determined on the teeth 16, 12, 24, 36, 32, and 44 using four-degree scales (from 0 to 3) [39]. The oral evaluation was done by two dentists (P.G. and K.G.), following nonstimulated (NWS) and stimulated (SWS) whole saliva sampling, and after previous calibration and training by an experienced dental specialist (A.Z.). The interexaminer and intraexaminer agreements for DMFT were evaluated by another dental examination in 10 patients after two weeks, with a *κ* that amounted to 0.96 and 1.00, respectively, whereas for PlI and GI *κ* were 0.96 and 0.92 and 0.92 and 0.96, respectively.

### 2.7. Biochemical Determination

All reagents (unless otherwise stated) were purchased from Sigma-Aldrich Nümbrecht, Germany, and Sigma-Aldrich Saint Louis, MO, USA. Salivary glycoxidation and nitrosative stress assays were performed in duplicate samples. The absorbance/fluorescence was measured using Infinite M200 PRO Multimode Microplate Reader (Tecan Group Ltd., Männedorf, Switzerland). The results were standardized to 1 mg of total protein (TP).

TP content was analyzed using a commercial kit (Thermo Scientific PIERCE BCA Protein Assay; Rockford, IL, USA), according to the manufacturer's instructions. The salivary amylase activity was determined colorimetrically using 3,5-dinitrosalicylic acid (POCH, Gliwice, Poland) as a substrate reaction [40]. The absorbance was measured at 540 nm.

### 2.8. Protein Glycation

The formation of salivary Amadori products was determined using nitro blue tetrazolium (NBT) assay [41]. The absorbance of samples was measured at 525 nm. The extinction coefficient of 12 640 cm^−1^ mol^−1^ l for monoformazan was used.

The content of salivary advanced glycation end products (AGE) was analyzed fluorimetrically at 350/440 nm [42]. Immediately before determination, saliva samples were diluted in 0.1 M H_2_SO_4_ (1 : 5, *v*/*v*) [27]. The characteristic fluorescence of pyraline, pentosidine, furyl-furanyl-imidazole (FFI), and carboxymethyl lysine (CML) was measured in 96-well black-bottom microplates.

### 2.9. Protein Oxidation

The concentration of salivary protein carbonyls (PC) was determined colorimetrically based on the reaction with 2,4-dinitrophenylhydrazine (2,4-DNPH) [43]. The absorbance was measured at 355 nm. The absorption coefficient of 22 000 cm^−1^ mol^−1^ l was used for calculations.

The concentration of total thiols was determined using Ellman's reagent [44]. The absorbance of samples was measured at 420 nm, and the concentration of total thiols was calculated from the calibration curve using reduced glutathione.

### 2.10. Protein Glycoxidation

The fluorescence assessment of salivary glycoxidative damage was also done. For this purpose, dityrosine, kynurenine, N-formylkynurenine, and tryptophan contents were measured fluorimetrically. The characteristic fluorescence at 330/415, 365/480, 325/434, and 295/340 nm, respectively, was measured in 96-well black-bottom microplates [45]. Immediately before determination, saliva samples were diluted in 0.1 M H_2_SO_4_ (1 : 5, *v*/*v*) [27]. The results were expressed in arbitrary fluorescence units (AFU)/mg protein.

### 2.11. Nitrosative Stress

The concentration of salivary nitric oxide (NO) was determined colorimetrically using sulfanilamide and N-(1-naphthyl)-ethylenediamine dihydrochloride [46, 47]. The absorbance was measured at 490 nm.

The salivary peroxynitrite concentration was determined colorimetrically based on peroxynitrite-mediated nitration resulting in nitrophenol formation [48]. The absorbance was measured at 320 nm.

The concentration of salivary nitrotyrosine was determined using ELISA commercial kit (Immundiagnostik AG; Bensheim, Germany), according to the manufacturer's instructions.

### 2.12. Statistics

GraphPad Prism 8.3.0 (GraphPad Software, Inc. La Jolla, USA) and Microsoft Excel 16.16.22 for macOS were used for statistical analysis. The distribution of results was assessed using the Shapiro–Wilk test, while homogeneity of variance using Levene's test. ANOVA analysis of variance and Tukey's HSD post hoc test were used for comparison of quantitative variables. ANOVA Kruskal–Wallis test and Dunn's post hoc test were used if the results' distribution was not normal. Multiplicity adjusted *p* value was also calculated. The nominal values were compared using the chi-square independence test. Correlations between salivary biomarkers were assessed using the Pearson correlation coefficient. The *p* value (two-tailed) was established as 0.05.

The number of patients was calculated *a priori* based on our preliminary study. Assuming the test power of 0.9 and *p* < 0.05, the minimum number of patients was 22 for each group (control and study). ClinCalc online calculator was used for the calculations.

## 3. Results

### 3.1. Clinical Characteristic

Sixteen stroke patients with normal salivary secretion, aged between 34 and 67 years, and fourteen individuals after stroke with hyposalivation, aged from 52 to 84 years, were recruited to the research. All subjects were in the subacute phase of cerebral stroke. The procedures, including oral examination and saliva sampling, were performed between 45 and 50 days following the stroke incident (on average 46.78 days; SD = 4.71). Table 1 presents detailed characteristics of both study groups and controls, with findings concerning demographic variables and habits of individuals.

### 3.2. Oral Health Status

Both groups of stroke patients presented the highest caries prevalence that amounted to 100.00%, which means none of the patients had DMFT = 0. The mean DMFT index in individuals with normal salivary secretion was lower than in persons with reduced salivary secretion (Table 2). In both groups of stroke patients, DMFT mainly depended on the number of missing teeth; however, individuals with HP had a much higher number of lost teeth than subjects with NS. In the stroke group with NS, only three individuals did not have active carious lesions (DT = 0), whereas in those with HP 9 subjects, while six patients were edentulous.

Both PlI and GI were higher in stroke patients with normal salivary secretion than in those with hyposalivation.

### 3.3. Salivary Gland Function

**Figure 1:**
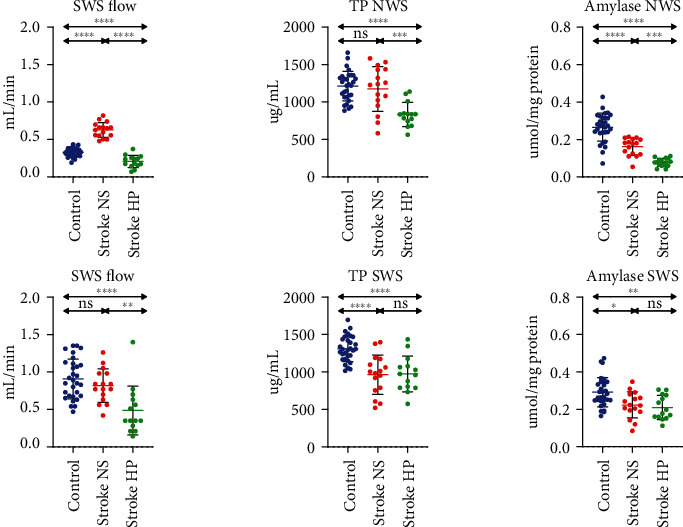
Salivary gland function in stroke patients and healthy controls. NS: stroke patients with normal salivary secretion; HP: stroke patients with reduced salivary secretion; NWS: nonstimulated whole saliva; SWS: stimulated whole saliva; TP: total protein concentration; ns: not significant. Differences statistically significant at ^∗^*p* < 0.05; ^∗∗^*p* < 0.005; ^∗∗∗^*p* < 0.0005; ^∗∗∗∗^*p* < 0.0001.

NWS's salivary flow was significantly higher in patients with stroke and normal salivary secretion than controls, while significantly lower in patients with stroke and hyposalivation. NWS's total protein content was considerably lower in patients with stroke and decreased salivary secretion than controls and stroke patients with normal salivation. The salivary amylase activity in NWS was significantly lower in both groups of stroke patients than controls and patients with stroke with hyposalivation compared to normal salivary output (Figure 1).

The stimulated salivary flow was significantly lower in patients with stroke and decreased salivary secretion than controls and patients with normal salivary function. Similarly, total protein content and salivary amylase activity in SWS were significantly lower in patients with stroke and decreased salivary secretion than controls and stroke patients with normal salivary function (Figure 1).

### 3.4. Protein Glycation

**Figure 2:**
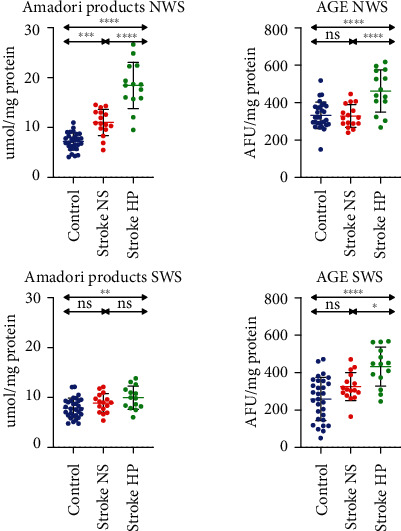
Protein glycation products in stroke patients and healthy controls. AGE: advanced glycation end products; NS: stroke patients with normal salivary secretion; HP: stroke patients with reduced salivary secretion; NWS: nonstimulated whole saliva; SWS: stimulated whole saliva; ns: not significant. Differences statistically significant at ^∗^*p* < 0.05; ^∗∗^*p* < 0.005; ^∗∗∗^*p* < 0.0005; ^∗∗∗∗^*p* < 0.0001.

The content of Amadori products was significantly higher in NWS for patients with stroke and reduced salivary secretion compared to patients with normal salivary flow and control. In SWS, Amadori products were significantly higher in the stroke HP group compared to the control (Figure 2).

In both nonstimulated and stimulated saliva in patients with stroke and reduced salivary secretion, the AGE content was significantly higher than patients with normal salivary flow and control (Figure 2).

### 3.5. Protein Oxidation

**Figure 3:**
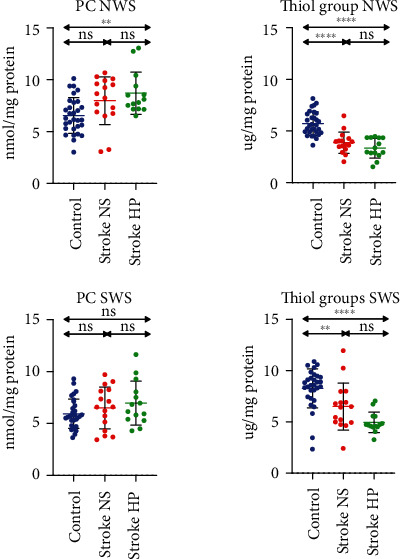
Protein oxidative damage in stroke patients and healthy controls. PC: protein carbonyls; NS: normal salivary secretion; HP: reduced salivary secretion; NWS: nonstimulated whole saliva; SWS: stimulated whole saliva; ns: not significant. Differences statistically significant at ^∗∗^*p* < 0.005; ^∗∗∗∗^*p* < 0.0001.

PC concentration was significantly higher in NWS of stroke patients with hyposalivation than controls, while in SWS, it did not differ between all groups (Figure 3).

In both NWS and SWS, the total thiols concentration was significantly lower in both groups of stroke patients than in control (Figure 3).

### 3.6. Protein Glycoxidation

**Figure 4:**
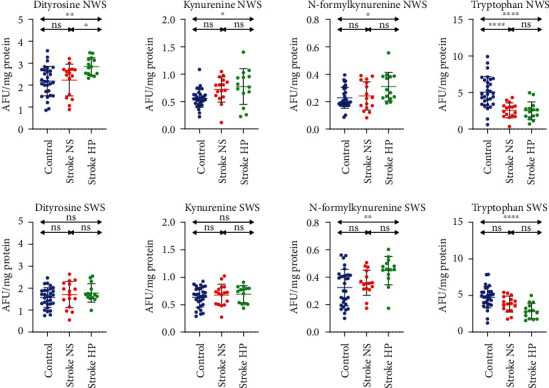
Glycoxidation products in stroke patients and healthy controls. NS: stroke patients with normal salivary secretion; HP: stroke patients with reduced salivary secretion; NWS: nonstimulated whole saliva; SWS: stimulated whole saliva; ns: not significant. Differences statistically significant at ^∗^*p* < 0.05; ^∗∗^*p* < 0.005; ^∗∗∗∗^*p* < 0.0001.

The fluorescence of oxidatively-modified amino acids was shown in Figure 4.

Dityrosine fluorescence was significantly higher in the NWS of patients with stroke and hyposalivation compared to other groups. In SWS, we did not show any statistical differences between the groups.

The fluorescence of kynurenine and N-formylkynurenine was significantly higher in NWS of stroke patients with hyposalivation than other groups. We found no significant differences in SWS.

Fluorescence of tryptophan was significantly lower in NWS and SWS of stroke patients with hyposalivation than controls and in NWS compared to stroke patients with normal salivary secretion.

### 3.7. Nitrosative Stress

**Figure 5:**
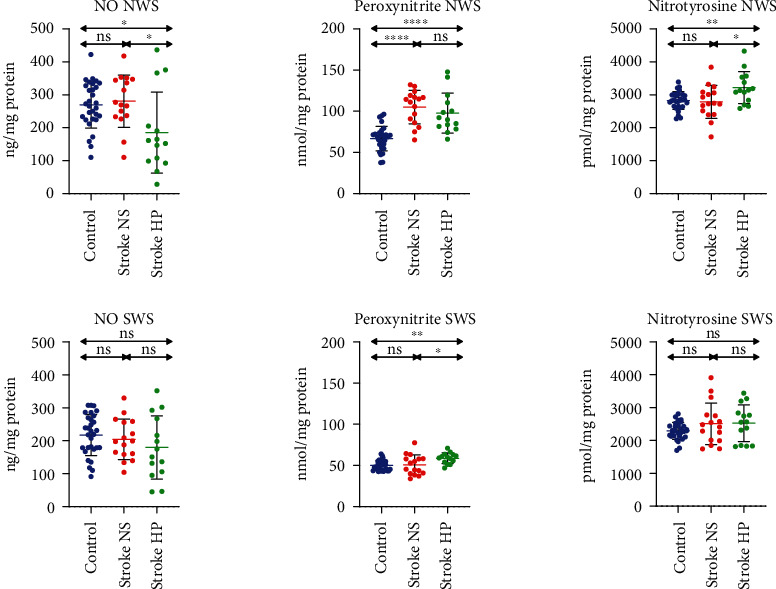
Nitrosative stress in stroke patients and healthy controls. NO: nitric oxide; NS: stroke patients with normal salivary secretion; HP: stroke patients with reduced salivary secretion; NWS: nonstimulated whole saliva; SWS: stimulated whole saliva; ns: not significant. Differences statistically significant at ^∗^*p* < 0.05; ^∗∗^*p* < 0.005; ^∗∗∗∗^*p* < 0.0001.

Salivary nitrosative stress was presented in Figure 5.

NO concentration was significantly lower in the group of stroke patients with hyposalivation compared to other groups. However, no differences in NO concentration in SWS were found.

The peroxynitrite content was significantly higher in NWS of both stroke groups compared to the control. In SWS, the ONOO^−^ content was considerably higher in patients with stroke and hyposalivation than stroke patients with normal salivary secretion and controls.

Nitrotyrosine concentration was significantly higher in patients with stroke and hyposalivation than patients with normal salivary secretion and control but did not differ considerably in SWS.

### 3.8. Comparison of NWS and SWS

In general, the content of glycoxidation products and nitrosative stress biomarkers was significantly higher in NWS compared to SWS in both control and stroke patients. However, total thiols were statistically higher in stimulated saliva compared to NWS. Interestingly, in stroke patients, Amadori products' concentration was significantly higher in NWS than SWS, and such changes were not observed in control (Table 3).

### 3.9. Correlations

In stroke patients, the content of glycoxidation products (Amadori products, AGE, PC, dityrosine, kynurenine, and N-formylkynurenine) in NWS correlated remarkably negatively with salivary flow rate, as did the concentration of selected nitrosative stress biomarkers (NO and nitrotyrosine). However, the content of total thiols correlated positively with the NWS flow. Such changes were not observed in SWS of stroke patients (except for dityrosine and N-formylkynurenine) as well as in NWS and SWS of healthy subjects (Table 4).

We did not show any significant correlations between salivary redox biomarkers and clinical parameters (data not shown).

## 4. Discussion

Saliva secretion is a reflex process. The reflex arch consists of afferent signals from sensory receptors in the oral cavity and is transmitted to the salivary nuclei in the medulla oblongata [49, 50]. However, in addition to afferent stimuli, the salivary nuclei also receive impulses from other brain centers. As a result, various neurotransmitters are released, which have a stimulating or inhibitory effect on salivary production [49–51]. Thus, in diseases of the central nervous system (CNS), salivary glands hypofunction and disturbances in protein secretion into saliva may occur. Since oxidative/nitrosative stress plays a crucial role in the pathogenesis of salivary gland dysfunction [12, 16, 22, 24, 52] as well as neurodegenerative diseases (including stroke) [4, 53, 54], we are the first to compare oxidation, glycation, and glycoxidation of proteins and nitrosative stress in stroke patients with normal salivary secretion and hyposalivation as well as with age and sex-matched control group.

The direct analysis of the formation of oxygen and nitrogen free radicals is practically impossible. Therefore, for the assessment of OS/nitrosative stress, the products of free radical interactions with cell components are most often used. They are much more durable than free radicals and provide information on the consequences of oxidative/nitrosative damage to the body [7].

We have shown increased oxidation and glycation of proteins in stroke patients with hyposalivation and enhanced nitrosative damage compared to stroke cases with normal salivary secretion and control groups. Interestingly, we found higher oxidative/nitrosative stress in nonstimulated saliva, which strongly correlates with salivary flow rate, total protein content, and salivary amylase activity. Notably, such relationships were not observed in the control groups.

Glycation is a slow physiological process that intensifies in many diseases [55]. Proteins with a high content of free amino acid groups, especially those containing lysine, are mainly glycated *in vivo*. Glycation is the formation of a bond between the free aldehyde group of glucose and the amino group of amino acid [55, 56]. The result is the creation of the labile Schiff base, which after a few weeks, converts into reactive Amadori products [57, 58]. Proteins that remain in the body for a longer time undergo further modifications (e.g., oxidation, dehydration, and fragmentation), resulting in the formation of advanced glycation end products (AGE) [59]. In our study, the content of protein glycation products (↑Amadori products and ↑AGE) was significantly higher in NWS of stroke patients with hyposalivation than patients with normal salivary secretion and healthy controls. However, in stimulated saliva, Amadori products, and AGE's content in these patients were significantly higher only in relation to control. Interestingly, AGE can combine with a specific receptor on salivary gland cells' surface, which triggers many proinflammatory signaling pathways such as NfkB, MAP-kinases, or p21RAS [16, 59]. There is an increased expression of many cytokines and chemokines under these conditions and an enhanced formation of oxygen/nitrogen free radicals [16, 24, 59]. Therefore, the glycation process is inextricably linked to the oxidation of proteins, which together are called glycoxidation. In our study, the severity of protein oxidation (↑PC and ↓total thiols) and glycoxidation (↑dityrosine, ↑kynurenine, ↑*N*-formylkynurenine, and ↓tryptophan) was significantly higher in NWS of stroke patients with hyposalivation compared to the control group. Products of protein oxidation/glycation can aggregate and accumulate in the salivary glands, leading to impaired secretory function and thus changes in saliva composition [14, 17–19]. This hypothesis may be confirmed by strong correlations between oxidation/protein glycation products and salivary flow rate, total protein content, and salivary amylase activity. Importantly, these changes were observed only in the NWS of stroke patients. Accumulation of such products as deposits may cause stiffening of the salivary glands' blood vessels/secretory cells. Furthermore, in these conditions, extracellular matrix hypertrophy can also occur [14, 17–19, 25]. Since it is ethically impossible to collect salivary gland samples from stroke patients with hyposalivation, further studies on an animal model are necessary to confirm our hypotheses. Nevertheless, OS-mediated salivary hypofunction has been demonstrated in patients with obesity [23, 24], hypertension [22], chronic kidney disease [16], chronic heart failure [25], but also neurodegenerative diseases such as dementia or Alzheimer's disease [27, 28]. Given that the risk factors for stroke are the abovementioned metabolic and cardiovascular diseases [1, 4], OS can lead to hypofunction of the salivary glands also in our patients.

The increase in salivary secretion in stroke patients with normosalivation (compared to control) may be surprising. However, this may suggest salivary secretion disorders at the level of extraganglionic parasympathetic and sympathetic nerves/salivary nuclei rather than at the salivary gland level [49, 50]. An increase in saliva production may also result from pharmacotherapy in the stroke's subacute phase [60]. Although the exact mechanism is unknown, the increase in saliva production can be attributed to the stimulation of muscarinic M4 and M1 receptors or to blocking the alpha 2-adrenergic receptor [61]. It should be remembered, however, that none of our patients have been found to have hypersalivation.

Nitric oxide (NO) plays an essential role in the initiation of saliva secretion [50, 62]. Indeed, NO produced by neuronal nitric oxide synthase (nNOS) (together with methacholine and substance P) leads to increased calcium ion concentration inside the secretory cells of salivary glands. This is responsible for activating the potassium and chloride channels dependent on Ca^2+^ and starts the formation of primary saliva [16, 62]. In our study, NO concentration in NWS was significantly reduced in stroke cases with hyposalivation compared to patients with normal salivary secretion and control. This suggests an impaired initiation of salivary secretion, which ultimately leads to hyposalivation in stroke patients. Reduced bioavailability of salivary NO is undoubtedly due to increased peroxynitrite production formed from NO and superoxide radical anions. ONOO- causes nitration of aromatic amino acids such as tryptophan and tyrosine and impairs many enzymes' functioning [63]. In our study, the content of ONOO^−^ and 3-nitrotyrosine was significantly higher in NWS of stroke patients with hyposalivation compared to healthy controls. Their content correlates strongly negatively with salivary glands' secretory function, indicating the role of nitrosative stress in salivary secretion impairment in stroke patients.

Saliva is produced in an amount of about 500-1000 ml per day. Without stimulation, the largest volume of saliva is produced by the submandibular glands. These salivary glands are mixed glands containing follicular cells, which secrete mucous and mucin-rich saliva. However, during stimulation, saliva is secreted mainly by the parotid salivary glands [35, 64, 65]. In contrast to NWS, the saliva produced by parotid glands is low-density, watery, and rich in *α*-amylase [65, 66]. In our study, increased oxidation/glycation of proteins and nitrosative stress was mainly observed in the nonstimulated saliva of stroke patients with hyposalivation. Moreover, the assessed redox biomarkers' content was significantly higher in NWS than in SWS, suggesting that mainly the submandibular salivary gland is disturbed in stroke patients. The consequence of reduced saliva secretion may be subjective dryness of the oral mucosa (xerostomia) and an increased incidence of dental caries, periodontal diseases, and fungal infections [14, 67]. Considering that antioxidant supplementation improves the secretory function of salivary glands in various systemic diseases [19, 68–70], the administration of free radical scavengers may be considered in stroke patients. Nevertheless, further studies are needed.

Nevertheless, salivary oxidative/nitrosative stress may not only be the origin of the salivary glands. In fact, many compounds pass from plasma to saliva through passive/facilitated diffusion and active transport or are also the gingival fluid filtrate [66, 71]. Unfortunately, in our study, we did not evaluate the products of protein glycation, oxidation and glycoxidation, and nitrosative stress in stroke patients' blood due to the lack of consent of the local ethics committee. However, we would like to point out that the evaluated redox biomarkers' concentration correlated remarkably negatively (Amadori, AGE, PC, dityrosine, kynurenine, N-formylkynurenine, and nitrotyrosine) or positively (total thiols and NO) with NWS flow rate only in stroke patients. This may indicate the involvement of OS/nitrosative protein damage in salivary gland hypofunction. The relationship between reduced salivary flow and enhanced oxidation, glycation, and nitration of proteins was observed in patients with obesity [23, 24], hypertension [22], chronic kidney disease [16, 72], and chronic heart failure [25]. Importantly, salivary oxidative stress did not correlate with changes at the central level (blood), and similar observations can be expected in stroke patients. Therefore, our study is the starting point for further clinical observations.

Many experimental and clinical studies have confirmed the contribution of OS/nitrosative stress in stroke pathology [9, 73, 74]. After cerebral damage resulting from ischemia/bleeding, posterior reperfusion occurs. Large amounts of oxygen are supplied to maintain the viability of the neurons. Nevertheless, at the same time, high ROS levels are generated, mainly in the mitochondrial respiratory chain. Since the mitochondria cannot keep up with the oxygen conversion, oxidative brain injury occurs [9, 73, 74]. The effect of ROS action is the peroxidation of membrane lipids, as well as the disturbed function of receptors, ion channels, and other cellular proteins [3, 4]. Although we have not evaluated the products of protein glycoxidation/nitration at a systemic level, it can be assumed that the common denominator of stroke complications in the brain and salivary glands may be oxidative/nitrosative stress. Unfortunately, our study does not explain why some patients with stroke develop hyposalivation, and some do not. Using correlation analysis, we did not show any relationship between demographic/clinical variables and redox status in stroke patients with hyposalivation and normal salivary secretion (data not shown). This issue undoubtedly requires further research and clinical observation, especially on a larger group of stroke patients.

It must be emphasized that the present research has some strengths. Firstly, the participants were adequately chosen from a stroke patient population and divided into two subgroups, i.e., normal and reduced salivary secretion. Secondly, two saliva samples from each individual, i.e., stimulated and unstimulated, were collected. Thirdly, saliva sampling was performed under the same conditions during the same season, and storage, handling, processing, and analysis methods were uniform. Finally, most subjects used the same diet, except for the individuals who had diabetes mellitus. Unfortunately, this study also has some limitations. We assessed only selected biomarkers of oxidative/nitrosative stress, and therefore, we could not fully characterize the redox homeostasis of stroke individuals. In addition, the redox status was determined only in saliva. Thus, in subsequent researches, it would be advisable to evaluate the saliva–blood interconnection of redox biomarkers and their diagnostic utility in much more numerous groups of subjects and including more biomarkers. Moreover, samples of saliva were collected from stroke individuals in the shortest possible time, i.e., in the disease's early subacute phase. The gathering of material directly after the stroke and receiving informed and written consent from all individuals was impossible because of patients' lesser or greater cognitive and physical deficits. Moreover, we could not exclude the influence of comorbidities on the assessed redox biomarkers. Finally, the impact of pharmacotherapy on the composition and secretion of saliva could not be eliminated.

## 5. Conclusion

In stroke patients, the function of salivary glands (mainly submandibular glands) is disturbed, and salivary protein glycoxidation/nitration is increased. Oxidative and nitrosative stress may be one of the mechanisms responsible for the impairment of saliva secretion in stroke patients. In these patients, antioxidant supplementation may be considered. However, extraglandular sources of salivary oxidative stress in stroke patients cannot be excluded. Further studies to assess salivary gland hypofunction in stroke cases are necessary.

## Figures and Tables

**Table 1:** Characteristics of both groups of stroke patients and controls.

Clinical characteristics		Control group *n* = 30	Study group *n* = 30		*p* value		
			Stroke with NS *n* = 16	Stroke with HP *n* = 14	NS vs. CG	HP vs. CG	NS vs. HP
Sex	Male *n* (%)	19 (63.33)	11 (68.75)	7 (50.00)	ns		
	Female *n* (%)	11 (36.67)	5 (31.25)	7 (50.00)			
Age in years	(mean ± SD)	62.47 ± 10.43	63.44 ± 7.74	61.36 ± 13.08	ns		
Place of residence	Urban Center *n* (%)	10 (33.33)	6 (37.5)	2 (14.28)	ns		
	Small town *n* (%)	9 (30.00)	4 (25.00)	7 (50.00)			
	Rural area or small village *n* (%)	11 (36.67)	6 (37.5)	5 (35.71)			
Household member(s)	With family member *n* (%)	18 (60.00)	11 (68.75)	10 (71.43)	ns		
	None *n* (%)	12 (40.00)	5 (31.25)	4 (28.57)			
Education	Primary *n* (%)	3 (10.00)	2 (12.5)	1 (7.14)	ns		
	Vocational *n* (%)	12 (40.00)	5 (31.25)	7 (50.00)			
	Secondary *n* (%)	7 (23.33)	3 (18.75)	4 (28.57)			
	University *n* (%)	8 (26.67)	6 (37.5)	2 (14.28)			
Systemic diseases	Hypertension *n* (%)	21 (70.00)	13 (81.25)	9 (64.29)	ns		
	Diabetes *n* (%)	8 (26.67)	4 (25.00)	4 (28.57)			
	Epilepsy *n* (%)	4 (13.33)	2 (12.5)	1 (7.14)			
	Arteriosclerosis *n* (%)	7 (23.33)	3 (18.75)	4 (28.57)			
	Gout *n* (%)	1 (3.33)	1 (6.25)	1 (7.14)			
	Limb thrombosis *n* (%)	2 (6.67)	2 (12.5)	0 (0.00)			
	Atrial fibrillation *n* (%)	3 (10.00)	1 (6.25)	3 (21.43)			
Medications	<5 drugs/day *n* (%)	10 (33.33)	7 (43.75)	5 (35.71)	ns		
	≥5 drugs/day *n* (%)	20 (66.67)	9 (56.25)	9 (64.29)			
Type of stroke	Hemorrhagic *n* (%)	—	3 (18.75)	3 (21.43)	ns		
	Ischemic *n* (%)	—	13 (81.25)	10 (71.43)			
	Ischemic ⟶ hemorrhagic *n* (%)	—	0 (0.00)	1 (7.14)			
Numbers of strokes in the patient's life	1	—	13 (81.25)	13 (92.86)	ns		
	2	—	3 (18.75)	1 (7.14)			
Cognitive and physical functional status	ACE III (mean ± SD)	97.27 ± 1.44	66.19 ± 27.03	72.93 ± 14.24	<0.0001	<0.0001	ns
	BI (mean ± SD)	20.00 ± 0.00	11.00 ± 4.21	10.57 ± 3.23	<0.0001	<0.0001	ns
	FIM (mean ± SD)	125.2 ± 0.71	87.44 ± 33.51	82.50 ± 29.22	<0.0001	<0.0001	ns
	BBS (mean ± SD)	55.53 ± 0.51	33.25 ± 15.91	27.64 ± 17.07	<0.0001	<0.0001	ns

Abbreviations: NS: normal salivary secretion; HP: reduced salivary secretion; ns: not significant; n: number of patients; CG: control group; ACE III: Addenbrooke's Cognitive Examination; BI: Barthel Index; FIM: functional independence measure; BBS: Berg Balance Scale; nd: no data; differences statistically significant at ^∗^*p* < 0.05; ^∗∗^*p* < 0.005; ^∗∗∗^*p* < 0.0005; ^∗∗∗∗^*p* < 0.0001.

**Table 2:** Dental characteristics of both groups of stroke patients and controls.

Dental characteristics	Control group *n* = 30	Study group (*n* = 30)		*p* value		
		Stroke with NS *n* = 16	Stroke with HP *n* = 14	NS vs. CG	HP vs. CG	NS vs. HP
DMFT (mean ± SD)	24.90 ± 7.60	22.63 ± 9.67	23.71 ± 3.31	ns	ns	ns
GI (in stroke patients with HP six completely edentulous patients were excluded from calculations, i.e., *n* = 8) (mean ± SD)	0.74 ± 0.79	0.87 ± 0.75	0.50 ± 0.65	ns	ns	ns
PlI (in stroke patients with HP six completely edentulous patients were excluded from calculations, i.e., *n* = 8) (mean ± SD)	1.28 ± 1.01	1.40 ± 0.93	1.06 ± 1.12	ns	ns	ns

Abbreviations: NS: normal salivary secretion; HP: reduced salivary secretion; ns: not significant; n: number of patients; CG: control group; DMFT index: a sum of decayed teeth (DT), teeth missing due to carious process (MT), and teeth filled because of caries (FT); GI: Gingival Index; PlI: Plaque Index.

**Table 3:** Comparison between NWS and SWS in healthy controls and stroke patients.

Parameter	Control			Stroke NS			Stroke HP		
	NWS	SWS	*p* value	NWS	SWS	*p* value	NWS	SWS	*p* value
Amadori products (*μ*mol/mg protein)	7.21 ± 1.69	7.89 ± 1.99	0.16	11.01 ± 2.6	8.89 ± 1.9	0.01	18.45 ± 4.7	9.96 ± 2.3	<0.0001
AGE (AFU/mg protein)	332.4 ± 70.75	259.1 ± 115	0.004	328.3 ± 60	324.8 ± 75	0.88	462 ± 113	432.3 ± 104	0.47
PC (nmol/mg protein)	6.55 ± 1.72	5.93 ± 1.41	0.13	7.98 ± 2.3	6.49 ± 2	0.06	8.70 ± 2	6.97 ± 2.1	0.04
Thiol groups (*μ*g/mg protein)	5.72 ± 1.13	8.27 ± 1.90	<0.0001	3.86 ± 1	6.50 ± 2.3	0.0002	3.34 ± 0.96	4.96 ± 0.99	0.0002
Dityrosine (AFU/mg protein)	2.19 ± 0.67	1.57 ± 0.45	<0.0001	2.23 ± 0.72	1.71 ± 0.60	0.03	2.85 ± 0.40	1.78 ± 0.42	<0.0001
Kynurenine (AFU/mg protein)	0.56 ± 0.16	0.64 ± 0.18	0.093	0.72 ± 0.23	0.68 ± 0.2	0.56	0.77 ± 0.33	0.69 ± 0.15	0.39
N-formylkynurenine (AFU/mg protein)	0.23 ± 0.07	0.32 ± 0.13	0.001	0.24 ± 0.1	0.36 ± 0.09	0.002	0.31 ± 0.1	0.45 ± 0.1	0.002
Tryptophan (AFU/mg protein)	5.05 ± 2.13	4.76 ± 1.47	0.55	2.57 ± 1	3.82 ± 1.1	0.002	2.50 ± 1.2	2.88 ± 1	0.39
NO (ng/mg protein)	269.3 ± 69.97	217.3 ± 62.4	0.004	280.7 ± 79	204.6 ± 62	0.005	185.5 ± 123	179.7 ± 96	0.89
Peroxynitrite (nmol/mg protein)	66.76 ± 14.84	50.12 ± 6.02	<0.0001	105 ± 20	50.72 ± 12	<0.0001	97.79 ± 24	58.74 ± 6.5	<0.0001
Nitrotyrosine (pmol/mg protein)	2825 ± 267.4	2293 ± 258.3	<0.0001	2783 ± 501	2509 ± 631	0.18	3220 ± 488	2529 ± 559	0.002

Abbreviations: NS: normal salivary secretion; HP: reduced salivary secretion; NWS: nonstimulated whole saliva; SWS: stimulated whole saliva; AGE: advanced glycation end products; PC: protein carbonyls; NO: nitric oxide.

**Table 4:** Correlations between clinical parameters, redox biomarkers, and salivary flow rate in healthy controls and stroke patients.

Parameter	Control				Stroke			
	*r* value	*p* value			*r* value		*p* value	
	NWS flow	SWS flow	NWS flow	SWS flow	NWS flow	SWS flow	NWS flow	SWS flow
Amadori products	0.197	-0.169	0.297	0.372	-0.875	0.084	<0.0001	0.658
AGE	0.255	0.03	0.174	0.873	-0.768	-0.283	<0.0001	0.13
PC	0.055	-0.394	0.772	0.031	-0.451	-0.268	0.012	0.153
Thiol groups	0.014	0.161	0.943	0.395	0.533	0.369	0.002	0.045
Dityrosine	-0.269	0.044	0.15	0.816	-0.706	-0.401	<0.0001	0.028
Kynurenine	0.096	0.353	0.613	0.055	-0.392	0.207	0.032	0.272
N-formylkynurenine	0.178	-0.167	0.348	0.378	-0.585	-0.484	0.001	0.007
Tryptophan	0.03	-0.102	0.876	0.592	0.291	0.155	0.119	0.414
NO	-0.344	-0.047	0.063	0.806	0.665	0.004	<0.0001	0.985
Peroxynitrite	-0.133	0.222	0.484	0.238	-0.102	0.025	0.592	0.898
Nitrotyrosine	0.028	-0.013	0.884	0.945	-0.682	0.072	<0.0001	0.706

Abbreviations: NS: normal salivary secretion; HP: reduced salivary secretion; NWS: nonstimulated whole saliva; SWS: stimulated whole saliva; AGE: advanced glycation end products; PC: protein carbonyls; NO: nitric oxide.

## Data Availability

The article contains complete data used to support the findings of this study.
